# Circulating levels of insulin-like growth factor-I (IGF-I) correlate with disease status in leprosy

**DOI:** 10.1186/1471-2334-11-339

**Published:** 2011-12-13

**Authors:** Luciana Silva Rodrigues, Mariana Andrea Hacker, Ximena Illarramendi, Maria Fernanda Miguens Castelar Pinheiro, José Augusto da Costa Nery, Euzenir Nunes Sarno, Maria Cristina Vidal Pessolani

**Affiliations:** 1 Laboratory of Cellular Microbiology, Instituto Oswaldo Cruz, Rio de Janeiro, RJ, Brazil; 2 Leprosy Laboratory, Instituto Oswaldo Cruz, Rio de Janeiro, RJ, Brazil; 3 Sérgio Franco Laboratory, Rio de Janeiro, RJ, Brazil

**Keywords:** Leprosy, IGF-I, IGFBP-3, Leprosy reactions, *Mycobacterium leprae*, Biomarker, Neuroendocrine system, Immune-inflammatory response

## Abstract

**Background:**

Caused by *Mycobacterium leprae *(ML), leprosy presents a strong immune-inflammatory component, whose status dictates both the clinical form of the disease and the occurrence of reactional episodes. Evidence has shown that, during the immune-inflammatory response to infection, the growth hormone/insulin-like growth factor-I (GH/IGF-I) plays a prominent regulatory role. However, in leprosy, little, if anything, is known about the interaction between the immune and neuroendocrine systems.

**Methods:**

In the present retrospective study, we measured the serum levels of IGF-I and IGBP-3, its major binding protein. These measurements were taken at diagnosis in nonreactional borderline tuberculoid (NR BT), borderline lepromatous (NR BL), and lepromatous (NR LL) leprosy patients in addition to healthy controls (HC). LL and BL patients who developed reaction during the course of the disease were also included in the study. The serum levels of IGF-I, IGFBP-3 and tumor necrosis factor-alpha (TNF-α) were evaluated at diagnosis and during development of reversal (RR) or erythema nodosum leprosum (ENL) reaction by the solid phase, enzyme-labeled, chemiluminescent-immunometric method.

**Results:**

The circulating IGF-I/IGFBP-3 levels showed significant differences according to disease status and occurrence of reactional episodes. At the time of leprosy diagnosis, significantly lower levels of circulating IGF-I/IGFBP-3 were found in NR BL and NR LL patients in contrast to NR BT patients and HCs. However, after treatment, serum IGF-I levels in BL/LL patients returned to normal. Notably, the levels of circulating IGF-I at diagnosis were low in 75% of patients who did not undergo ENL during treatment (NR LL patients) in opposition to the normal levels observed in those who suffered ENL during treatment (R LL patients). Nonetheless, during ENL episodes, the levels observed in RLL sera tended to decrease, attaining similar levels to those found in NR LL patients. Interestingly, IGF-I behaved contrary to what was observed during RR episodes in R BL patients.

**Conclusions:**

Our data revealed important alterations in the IGF system in relation to the status of the host immune-inflammatory response to ML while at the same time pointing to the circulating IGF-I/IGFBP-3 levels as possible predictive biomarkers for ENL in LL patients at diagnosis.

## Background

Leprosy, a chronic infectious disease caused by the obligate intracellular bacterium *Mycobacterium leprae *(ML), remains a major source of morbidity in developing countries [1]. The disease principally affects the skin and peripheral nervous system in which the leprosy bacillus is preferentially found inside macrophages and Schwann cells (SC) [2]. This tissue tropism causes nerve damage, which, in turn, leads to sensorial impairment and permanent disabilities, by far the major health concerns facing leprosy patients today.

Also known as Hansen's disease, leprosy manifests as a spectrum of clinical forms in correlation with the nature and magnitude of the innate and adaptive immune responses generated during ML infection [3]. At one extreme of the spectrum, individuals with tuberculoid leprosy (TT) have few lesions and manifest a contained, self-limited infection in which scarce bacilli are detected due to the generation of a strong cellular immune response against ML. At the other, lepromatous leprosy (LL) is a progressively disseminating disease characterized by extensive bacterial multiplication within host cells and low cell-mediated immunity (CMI) to the pathogen. Between these two poles are the borderline forms (characterized by their intermediate clinical and immunological patterns), commonly referred to as borderline tuberculoid (BT), borderline borderline (BB), and borderline lepromatous (BL) in accordance with their proximity to either one of the spectral extremes.

Nerve damage occurs in all clinical forms of the disease and may progress during multidrug therapy (MDT) itself and even subsequent to patient release, due, for the most part, to the occurrence of acute immune-inflammatory episodes known as leprosy reactions. The most frequent leprosy reactions are classified as Type 1 (reversal) reaction (RR) and Type 2 reaction, or erythema nodosum leprosum (ENL). Many patients have been known to experience recurrent episodes [4]. Neuritis and cutaneous inflammation are prominent symptoms of both types of reaction with systemic repercussions, as seen in the occurrence of malaise, fever, and inflammation in other tissues. RR predominates in BL patients with a bacilloscopic index (BI) below 3 while ENL occurs in LL patients with high BI [5,6]. Over the past few decades, numerous studies have been conducted, enhancing our knowledge of the epidemiological, clinical, and laboratory risk factors for neuropathy and reaction in leprosy. However, our understanding of the physiopathology of reaction remains limited so that further research is urgently needed to more clearly define laboratory biomarkers capable of accurately identifying the leprosy patients most at risk of developing reaction.

The interaction between the immune and neuroendocrine systems plays a crucial role in host homeostasis during the adaptive response to stress and infection [7,8]. Indeed, an increasing understanding of how inflammation is held under control by the neuroendocrine system has greatly contributed to our knowledge of the physiopathology of several immune-inflammatory illnesses. Furthermore, credible evidence has shown that, during the immune-inflammatory response to infection, the GH/IGF-I/IGFBP-3 somatotropic axis exercises a prominent regulatory role [9]. These hormones not only affect cellular metabolism but are likewise able to interact with cytokines and glucocorticoids (GCs) in modulating the immune-inflammatory response [10]. Insulin-like growth factor-I (IGF-I) circulates in relatively high concentration levels in plasma (150-400 ng/mL), which varies according to age [11]. Insulin-like growth factor binding protein-3 (IGFBP-3) binds to 80-90% of circulating IGF in a stable ternary complex with an acid-labile subunit (ALS) serving as the main reservoir for plasma IGF-I [12]. Most of the IGF-I found in circulation is produced in the liver under growth hormone (GH) regulation.

Unfortunately, little, if anything, is known about the interaction between the immune and neuroendocrine systems in leprosy [13]. Since the disease has a strong immune-inflammatory component, it is hypothesized that the cross-talk taking place between these two systems may have a profound effect on the natural course of ML infection. In the present investigation, a retrospective study was conducted to compare the circulating levels of IGF-I and IGFBP-3 across the spectral clinical forms of leprosy in conjunction with reactional vs. nonreactional LL and BL patients both at diagnosis and during reaction. Our results demonstrate significant differences in circulating IGF-I/IGBP-3 in leprosy according to disease status, indicating that these proteins could be used to identify patients at high risk of reaction.

## Methods

### Patients and samples

The study population consisted of 21 healthy controls (HC) from endemic laboratory staff volunteers (3 males, 18 females, ranging in age from 19 to 62) and 93 patients (50 males, 43 females, in a 18-65 age range) referred to the Souza Araújo Ambulatory (Reference Center for Leprosy Diagnosis and Treatment, Oswaldo Cruz Foundation, Rio de Janeiro, RJ, Brazil) for the diagnosis and treatment of leprosy. Healthy controls and patients were carefully screened by anthropometric measurements (body mass index, BMI) and biochemical analysis; and those with diabetes, obesity or cholesterol disorders were excluded from the study. Each patient was assessed clinically throughout treatment and detailed analyses of medical and dermatological examinations were routinely carried out. Bacteriological examinations of slit-skin smears were performed to determine BI. The patients were categorized according to the Ridley and Jopling's classification scale [3] into borderline tuberculoid (BT), borderline lepromatous (BL), or lepromatous (LL). The diagnosis of leprosy Type 1 reaction (RR) was determined by the appearance of erythema and edema in either existing or new skin lesions. The diagnosis of leprosy Type 2 reaction (ENL) was primarily based on the acute occurrence of nodular skin lesions, accompanied by fever with or without peripheral nerve pain and/or nerve dysfunction. Nonreactional (NR) groups were selected among leprosy patients who were rendered the same histopathological classification as reactional (R) patients but who did not experience reaction at initial diagnosis or during follow-up. These NR patients were distributed into the following groups: 13 nonreactional BT (NR BT); 19 nonreactional BL (NR BL); 18 nonreactional LL (NR LL) patients, 25 BL who had RR during treatment (R BL); and 18 LL who developed ENL during treatment (R LL). Blood was collected at three different time points: i) at the beginning of leprosy treatment, ii) at diagnosis of a reactional episode if one occurred; and iii) 3-5 years post-treatment. Blood samples were taken from 1995 to 2005; and patient sera were extracted and stored at -20°C until retrieval for analysis. The baseline characteristics of each group of individuals included in the study are shown in Table 1.

**Table 1:** Baseline characteristics of leprosy patients and healthy controls

Characteristics	HC	NR BT	NR BL	R BL	NR LL	R LL
Individuals (n)	21	13	19	25	18	18
Sex						
Male	03	07	11	14	05	13
Female	18	06	08	11	13	05
Age (median)	36.9	35.8	42.7	39.4	37.1	37.2
(min-max)	(19-63)	(22-65)	(19-64)	(18-68)	(20-62)	(21-65)
Bacilloscopic Index						
(median)	-	0.08	1.78	1.89	4.0	4.7

Groups included in this study: HC, healthy controls; NR BT, nonreactional borderline tuberculoid patients; NR BL, nonreactional borderline lepromatous patients; R BL, reactional borderline lepromatous patients; NR LL, nonreactional lepromatous patients; R LL, reactional lepromatous patients

Patients received World Health Organization-recommended leprosy multidrug therapy (MDT). Reactions were treated with prednisolone, starting at 40 mg/day, and gradually tapering off over a 12-week period.

### Ethical considerations

This study was approved by the Ethics Committee of the Oswaldo Cruz Foundation. Informed written consent was obtained from all patients or their guardians and the healthy controls prior to specimen collection.

### IGF-I, IGFBP-3, and TNF-α measurement

The serum levels of IGF-I, IGFBP-3, and TNF-α were measured by the IMMULITE 2000 Analyzer (EuroDPC Med Limited, Llanberis, UK). Assays were carried out using the solid phase, enzyme-labeled, chemiluminescent-immunometric method in accordance with the manufacturer's instructions. IGF-I and IGFBP-3 levels were considered normal according to the reference range by age provided by the kit manufacturer [14].

### Statistical analysis

Data are expressed as median, mean ± S.D. values. Group comparisons were evaluated by the analysis of a variance (ANOVA) test, using age as a covariate, followed by Bonferroni's test for multiple comparisons. Paired *t *test was employed to compare IGF-I, IGFBP3, and TNF-α levels before and during reaction. Pearson partial correlation coefficient (controlled for age) was calculated between BI and IGF-I and IGFBP-3 levels. All statistical calculations were done via the SPSS software program; and *p *values lower than 0.05 were considered statistically significant.

## Results

### Lepromatous patients showed below-normal levels of serum IGF-I during active disease

**Figure 1:**
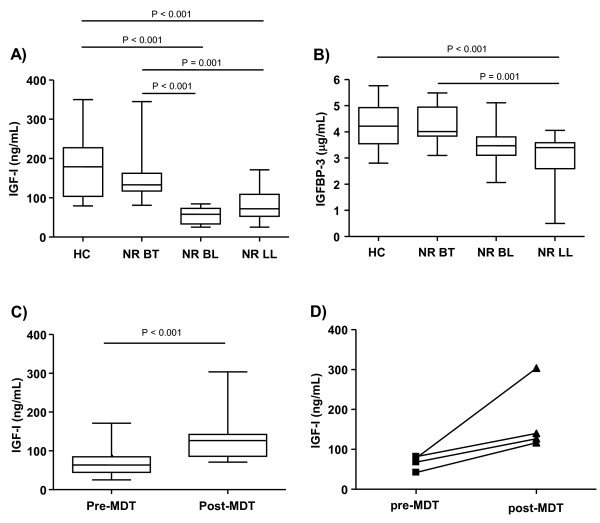
**Serum IGF-I and IGFBP-3 levels along the spectrum of leprosy clinical forms**. Box-plots represent the serum levels of IGF-I (**a**) and IGFBP-3 (**b**) assessed in healthy controls (HC) and nonreactional borderline tuberculoid patients (NR BT), borderline lepromatous leprosy patients (NR BL), and lepromatous leprosy patients (NR LL) prior to MDT. (**c**) Serum levels of IGF-I of NR BL/LL patients pre-MDT and post-MDT. Median values are indicated by lines (-). Statistical differences between the groups were evaluated by ANOVA, using age as a covariate. (**d**) Represent the circulating levels of IGF-I in paired pre- and post-MDT serum samples from the same patient. Each line represents one patient.

Evidence in the literature has shown that the somatotropic axis is responsible for modulating the immune system, directly influencing both the humoral and cellular immune (CMI) responses [15,16]. Since the spectral clinical forms of leprosy occur as a result of the capacity of the host to mount anywhere from low- (LL)-to-high (TT) CMI responses against ML, the serum levels of IGF-I and IGFBP-3 were compared among patients having the different clinical forms at the pre-MDT stage. Only those who did not undergo reactional episodes during the course of the disease were included in this analysis, and the levels found in leprosy patients were compared with those of the HCs. The IGF-I levels were significantly lower in NR BL and NR LL patients when compared to the NR BT (*P *= 0.001) and HC (*P *< 0.001) groups (Figure 1a). A decrease in IGFBP-3 levels was also observed in NR LL patients when compared to the NR BT (*P *< 0.001) and HC (*P *= 0.001) individuals (Figure 1b). According to the reference range by age, 83,3% (10/12) and 75% (12/16) of the BL and LL patients, respectively, showed below-normal levels of serum IGF-I. Moreover, via Pearson's partial correlation analysis, a weak inverse correlation was found between elevated BI and reduced IGF-I (r = -0.56, *P *< 0.001) and IGFBP-3 (r = -0.47, *P *< 0.001) levels in these patients (data not shown).

### Serum IGF-I in lepromatous patients increased reaching normal levels after treatment

Next, to reinforce the notion that the low IGF-I levels observed in the LL patients resulted from active modulation by ML infection, their hormone levels were measured at the conclusion of MDT. Interestingly, when serum IGF-I was evaluated in a group of nonreactional BL/LL patients 3-5 years post-MDT treatment (n = 9), significantly higher levels (*P *< 0.001) were observed in comparison to those found in the group evaluated at diagnosis (n = 28) (Figure 1c), with 78% of this post-MDT nonreactional BL/LL fitting into the normal range. Increment of serum IGF-I levels after treatment can be clearly seen in paired pre- and post-MDT serum samples taken from the same patient (Figure 1d).

### Circulating IGF-I and IGFBP-3 levels before and during ENL episodes

Since the somatrotropic axis plays an important regulatory role during host immune-inflammatory responses to infection, we then investigated circulating IGF-I and IGFBP-3 levels in the context of ENL episodes. This type of inflammatory episode occurs principally in LL patients [6,17], the main concern being the management of leprosy care to prevent disabilities [18]. For this purpose, a group of LL patients who suffered ENL episodes during MDT (referred to as R LL; n = 18) were enrolled in the study. Serum samples of the R LL patients were obtained at two different time points: i) at the onset of MDT, at which time no signs of reaction were detected (referred to as R LL_t = 0_); and ii) during reactions (referred to as R LL_ENL_) taking place between 3 weeks and 2 years after the beginning of treatment (mean average of 11.5 ± 9.3 months).

**Figure 2:**
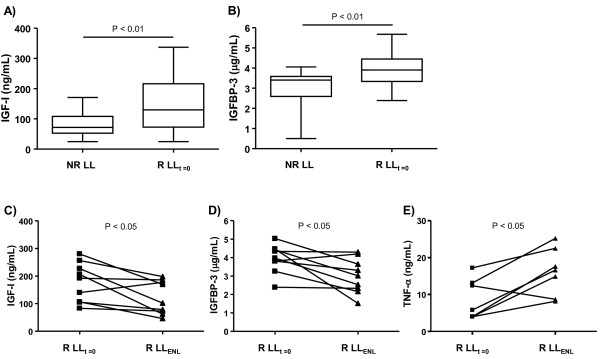
**Circulating IGF-I and IGFBP-3 levels change according to disease status in LL patients**. Box-plots represent the serum levels of IGF-I (**a**) and IGFBP-3 (**b**) assessed in nonreactional (NR LL) and reactional LL (R LL_t = 0_) patients at the pre-MDT stage. Median values are indicated by lines (-). (**c**), (**d**) and (**e**) represent the serum levels of IGF-I, IGFBP-3, and TNF-α, respectively, at the pre-MDT stage and during ENL (R LL_ENL_). Each line represents one patient. Paired t test was used for statistical analysis.

It was first investigated whether reactional LL patients (clinically unstable patients) already display distinct levels of IGF-I and IGFBP-3 at diagnosis in comparison to nonreactional LL patients (a clinically stable group). Notably, the R LL_t = 0 _samples presented significantly higher levels of IGF-I and IGFBP-3 than the NR LL group (*P *< 0.01), as can be seen in Figure 2a, b, respectively. The behavior of these proteins was subsequently monitored in the R LL group during ENL. Interestingly, these levels decreased dramatically (R LL_ENL_; *P *< 0.05) during reaction, reaching values similar to those detected in the NR LL group, as observed in Figure 2c, d.

TNF-α was also quantified in the same serum samples due to the well-established role of this pro-inflammatory cytokine in ENL [19,20] along with its antagonistic activity in IGF-I [21]. Similar range levels of TNF-α were observed in NR LL and R LL_t = 0 _serum samples (data not shown). However, consistent with previous published data [19,20], TNF-α levels were higher during ENL (*P *< 0.05) in comparison to those measured prior to reaction (Figure 2e). Taken together, these results demonstrate that the IGF-I and IGFBP-3 circulating levels at disease diagnosis were significantly higher in the LL patients who developed ENL than among the LL patients who did not experience reaction during MDT. In the course of ENL episodes, however, these levels tended to decrease, attaining levels similar to those found among the NR LL patients.

### Circulating IGF-I and IGFBP-3 levels before and during RR episodes

Next, the IGF-I and IGFBP-3 levels in the context of RR episodes, another frequent type of acute inflammatory episode in leprosy, were investigated. Type 1 reaction, or RR, more common among BL patients, affects approximately one-third of these patients, especially in the first year of MDT [22]. For this reason, a group of BL who underwent RR episodes during MDT (referred to as R BL; n = 25) was included in this analysis. Serum samples were obtained at 2 different time points in R BL patients: i) at the onset of MDT, when no signs of reaction were detected (referred to as R BL_t = 0_); and ii) during reaction (referred to as R BL_RR_) occurring between 2 weeks and 18 months after the beginning of treatment (mean average of 6.6 ± 4.8 months).

**Figure 3:**
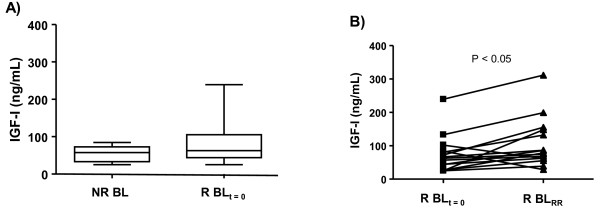
**Circulating IGF-I and IGFBP-3 levels change according to disease status in BL patients**. **a**. The box-plot represents the serum levels of IGF-I assessed in nonreactional (NR BL) and reactional BL (R BL_t = 0_) patients at the pre-MDT stage. Median values are indicated by lines (-). **b**. Represent the serum levels of IGF-I at the pre-MDT stage and during RR (R BL_RR_). Each line represents one patient. Paired t test was used for statistical analysis.

As a first step, circulating levels of IGF-I and IGFBP-3 in R BL vs. NR BL patients at disease diagnosis were compared. In contrast to the differences observed for LL patients in the context of ENL, similar levels of IGF-I and IGFBP-3 were detected in NR BL and R BL patients (Figure 3a; data not shown). The next step involved measuring the levels of IGF-I and IGFBP-3 before and during RR episodes in R BL patients (n = 15). Curiously, in contrast to the decreasing levels observed during ENL, IGF-I levels significantly increased during RR (*P *< 0.05), as shown by the individual behavior of each patient displayed in Figure 3b. However, no significant alterations were observed in IGFBP-3 and TNF-α levels during RR (data not shown).

## Discussion and Conclusions

The interplay between the immune and neuroendocrine systems exerts a critical role in the maintenance of host homeostasis during infection. The neuroendocrine system not only favors the building of an effective immune response against the pathogen, but also controls its intensity, thus avoiding extensive tissue damage [7]. Leprosy is an infectious disease with a strong immune-inflammatory component, whose status dictates both the clinical form of the disease as well as the occurrence of reactional episodes. This particular characteristic makes this disease an especially attractive model for studying neuroimmunoendocrine interactions in that it allows for a broader understanding of the interplay among hormones, cytokines, and GCs during chronic inflammation in combination with acute inflammatory episodes in response to mycobacterial antigens. One of the many players involved in this interaction is the hormone IGF-I, previously shown to suffer a serious imbalance during infectious and inflammatory diseases [7,9]. In the present study, for the first time the IGF system was analyzed during the course of leprosy. Analysis was done by measuring the circulating levels of IGF-I and IGFBP-3 in patients representing status variations in the immune-inflammatory response to ML infection.

Circulating IGF-I/IGFBP-3 was initially examined across the leprosy spectrum. Interestingly, while no significant difference was observed between BT patients and HC, both the nonreactional BL and LL (multibacillary) patients showed low levels of IGF-I/IGFBP-3. It is worth noting that the IGF-I/IGFBP-3 levels across the leprosy spectrum appeared to follow a trend similar to the one observed with respect to serum IFN-γ [23,24] (higher at the tuberculoid pole) but contrary to what was found regarding the circulating levels of the pro-inflammatory cytokines IL-1-β, IL-6, and TNF-α (higher at the LL pole) [19,20]. Moreover, recovery of IGF-I levels after treatment suggests that the low levels observed in these patients resulted from a direct down modulation by ML infection.

The low circulating levels of IGF-I/IGFBP-3 found in nonreactional LL patients (~75% with below-normal levels) were in parallel with those found in other critical illnesses like sepsis [25,26]. It is known that the inflammatory response to sepsis is followed by the development of a hypo-inflammatory and impaired immune response, which is unable to eradicate the infection [27]. The low IGF-I/IGFBP-3 levels found in the chronic phase of sepsis are the result of overactivation of the hypothalamic-pituitary-adrenal (HPA) axis by the excessive and prolonged production of pro-inflammatory cytokines, leading to the subsequent peripheral secretion of GCs and inhibition of IGF synthesis by the liver [7]. Interestingly, in tuberculosis, another mycobacterial disease, no alterations in circulating IGF-I levels were observed when patients with different degrees of pulmonary involvement and healthy controls were compared [28].

LL patients and sepsis share this immunosuppressive state. In polar LL patients, the absence of a CMI response against ML allows the pathogen to proliferate indiscriminately, reach high numbers, and disseminate systemically throughout the bloodstream. Although presenting high bacteremia, approximately 50% of LL patients are clinically stable, which can be considered clear evidence of a controlled, finely-regulated immune-inflammatory response due to the activation of anti-inflammatory loops that prevent over-inflammation and subsequent immune-mediated tissue damage. It is, therefore, reasonable to speculate that the HPA axis, assumed to be the main physiological feedback loop in inflammation, is activated in these patients, playing a critical role in maintaining homeostasis. HPA axis activation in LL patients is expected, based on the high circulating levels of the pro-inflammatory cytokines detected such as IL-1β, IL-6, and TNF-α [19,20]. To date, however, data concerning the circulating levels of cortisol and the adrenal functional status in leprosy have been conflicting [13]. Moreover, intrinsic anti-inflammatory mechanisms such as the high production of IL-10 observed in these patients [29] may complement the immune-suppressive effects of GCs, making possible a controlled immune-inflammatory response in a scenario of hyper-stimulation of the host immune system resulting from accumulated concentrations of mycobacterial antigens.

It is noteworthy that the LL patients who had ENL during treatment showed significantly higher levels of IGF-I/IGFBP-3 at the pre-MDT stage (up to 2 years before reaction), suggesting that changes in these proteins may reflect the delicate balance between the pro- and anti-inflammatory responses in these patients and, consequently, the risk of initiating an uncontrollable inflammatory event. Again, in these patients, the higher IGF-I/IGFBP-3 levels found among R LL patients may be the result of insufficient anti-inflammatory feedback, necessary for the maintenance of homeostasis in the presence of high concentrations of ML antigens. While lower levels of IGF-I/IGFBP-3 might be reflective of high immune-suppression levels, a controlled immune-inflammatory response, and high clinical stability in NR LL patients, higher levels of this hormone in R LL patients might indicate reduction of suppression and ENL development.

Our results are consistent with previous data indicating high systemic production of TNF-α during ENL [19]. Also observed was a reduction in IGF-I/IGFBP-3 levels during ENL. This finding could be interpreted as an attempt to reach the low levels observed in NR LL, most likely the result of the activation of an anti-inflammatory loop such as the release of GCs in response to the presence of high levels of pro-inflammatory cytokines produced during reaction.

It should be highlighted that whenever a group of BL patients was analyzed, interesting but opposing findings were observed according to the immunological stability of the patients in the context of changes in circulating IGF-I/IGFBP-3. In contrast to the LL group undergoing or not ENL, no differences in circulating IGF-I/IGFBP-3 between nonreactional vs. reactional BL (who underwent RR during treatment) at the time of diagnosis were observed. Moreover, in contrast to ENL, during RR, an increase in IGF-I/IGFBP-3 was demonstrated while, consistent with previous data [30,31], no changes in the TNF-α serum levels were detected. Differently from LL patients, however, BL patients are known to build a weak CMI response against ML [23,24]; and RR is characterized by an increase in the Th1 response to ML antigens, which is capable of rapidly triggering nerve damage [32]. Based on the capacity of IGF-I to stimulate the secretion of IL-10 by activated T cells [16], it is tempting to speculate an anti-inflammatory role for this hormone in BL patients by its ability to dampen local inflammation in the skin and nerves in nonreactional patients and during RR. Thus, increased IGF-I levels during RR could be interpreted as an attempt to re-establish homeostasis.

In conclusion, our data revealed important alterations in the IGF system in relation to the status of the host immune-inflammatory response to ML. These findings support the hypothesis that interactions between the immune and neuroendocrine systems play a critical role during the natural course of ML infection, contributing to a controlled immune-inflammatory response across the spectral clinical forms of leprosy. Disruption of immune neuroendocrine homeostasis seems to be associated with acute, uncontrolled inflammatory episodes. Of note, our data indicate that circulating IGF-I and IGFBP-3 levels can be reliably used as predictive biomarkers for ENL at diagnosis, favoring the development of new strategies for the management of leprosy patients and the prevention of reaction and neuropathy.

## List of abbreviations

BB: Borderline borderline leprosy; BI: Bacilloscopic index; BL: Borderline lepromatous leprosy; BT: Borderline tuberculoid leprosy; CMI: Cell-mediated immunity; CNS: Central nervous system; ENL: Erythema nodosum leprosum; GCs: Glucocorticoids; GH: Growth hormone; HC: Healthy control; HPA: Hypothalamic-pituitary-adrenal axis; IGFBP-3: Insulin-like growth factor binding protein-3; IGF-I: Insulin-like growth factor-I; LL: Lepromatous leprosy; MDT: Multidrug therapy; ML: *Mycobacterium leprae; *NR BL: Nonreactional BL; NR BT: Nonreactional BT; NR LL: Nonreactional LL; PBMC: Peripheral blood mononuclear cells; PNS: Peripheral nervous system; R BL: Reactional BL; R LL: Reactional LL; RR: Reversal reaction; SC: Schwann cells; TNF-α: Tumor necrosis factor-alpha; TT: Tuberculoid leprosy.

## Competing interests

The authors declare that they have no competing interests.

## Authors' contributions

LSR recruited patients, designed the study, performed the measurements, analyzed the data, contributed with reagents/material, and wrote the paper. MAH analyzed the data. XI recruited patients and also analyzed the data. MFMCP performed the measurements and contributed with reagents/material. JACN recruited patients. ENS recruited patients, contributed with reagents/material/analysis tools, and wrote the paper. MCVP conceived and designed the study, coordinated the field work, analyzed the data, contributed with reagents/material, and wrote the paper. All authors read and approved the final manuscript.

## Pre-publication history

The pre-publication history for this paper can be accessed here:

http://www.biomedcentral.com/1471-2334/11/339/prepub
